# Cancer-associated fibroblasts-derived gamma-glutamyltransferase 5 promotes tumor growth and drug resistance in lung adenocarcinoma

**DOI:** 10.18632/aging.103429

**Published:** 2020-07-08

**Authors:** Jia-Ru Wei, Jun Dong, Lei Li

**Affiliations:** 1 State Key Laboratory of Ophthalmology, Zhongshan Ophthalmic Center, Sun Yat-Sen University, Guangzhou, P. R. China; 2 State Key Laboratory of Oncology in South China and Collaborative Innovation Center for Cancer Medicine, Sun Yat-Sen University Cancer Center, Guangzhou, P. R. China; 3 Department of Clinical Oncology Center, The University of Hongkong-Shenzhen Hospital, Shenzhen, P. R. China; 4 Department of Clinical Oncology, The University of Hong Kong, Hong Kong, P. R. China

**Keywords:** GGT5, cancer-associated fibroblast, lung adenocarcinoma, glutathione, ROS

## Abstract

Gamma-glutamyltransferase 5 (GGT5) is a member of the gamma-glutamyl transpeptidase gene family with the capacity of cleaving the gamma-glutamyl moiety of glutathione, but its role in cancer progression has never been revealed. In this study, we found that gene *GGT5* was highly expressed in cancer-associated fibroblasts (CAFs) in lung adenocarcinoma, predicting the unfavorable survival of patients with lung adenocarcinoma. Cell growth, foci formation and spheres formation analyses showed that cancer cell proliferation was attenuated under treatment with the conditioned media from GGT5-silenced CAFs. Moreover, high expression of GGT5 in CAFs enhanced the drug resistance of cancer cells by increasing intracellular glutathione and reducing the intracellular reactive oxygen species in cancer cells. In mouse xenograft model, we proved that targeting GGT5 with a small-molecule inhibitor GGsTop could inhibit tumor growth and increase the chemosensitivity of cancer cells. Taken together, our study illuminates that high level of GGT5 in CAFs contributes to cancer cell survival and drug resistance, indicating that GGT5 may be a promising therapeutic target in lung adenocarcinoma.

## INTRODUCTION

Lung cancer is a highly aggressive neoplasm with an unfavorable survival [1]. Non-small cell lung cancer (NSCLC) is the most common histologic type of lung cancer, accounting for about 85% of all cases. Now, the 5-year survival rate is only 10-15% for NSCLC patients in advanced stage [2]. Lung squamous cell carcinoma (LUSC) and lung adenocarcinoma (LUAD) are two main pathological types of NSCLC. LUSC is closely correlated with a history of tobacco smoking. LUAD is currently the most common type of NSCLC in lifelong non-smokers, accounting for approximately 40% of NSCLC cases [1, 3]. However, the overall survival of patients with LUAD is poorer than LUSC patients, which may be the result of the different pathogenesis, tumor microenvironment or aberrant oncogenes [4].

Gamma-glutamyltransferase (GGT) is a membrane-bound extracellular enzyme involved in the hydrolysis of gamma-glutamyl bonds of glutathione (GSH) and other gamma-glutamyl compounds. In animals, GGT is highly expressed in tissues with secretory or absorptive functions, such as kidney, intestine, liver, prostate and gallbladder [5]. Moreover, the enzyme GGT as a new non-invasive marker can be detected in blood, which is used to indicate many diseases. Recent studies have demonstrated that increased serum GGT level predicted the advanced histological hepatic injury, insulin resistance and other chronic disease [6, 7]. Elevated serum GGT was associated with the ventricular repolarization abnormalities in the early stage of T2DM [8]. In addition, most studies showed that GGT expression was often significantly increased in human malignancies. For instance, overall survival for gallbladder cancer patients with elevated GGT was significantly worse than patients with the normal level of GGT [9]. Patients with primary metastatic breast cancer in the high risk GGT group had a poorer overall survival, compared to the low risk group [10]. Moreover, high pre-therapeutic GGT serum levels was associated with the advanced tumor stage and could serve as an independent prognostic marker for the worse survival in patients with epithelial ovarian cancer [11], pancreatic cancer [12] and endometrial cancer [13]. These studies just focus on the prognostic role of GGT in cancers, but its functions and mechanisms during cancer progression are not fully disclosed.

Gamma-glutamyltransferase 5 (GGT5), also known as GGT-like activity 1 (GGTLA1), is a member of the GGT protein family. Many tissue-local macrophages, such as Kupffer cells in the liver and alveolar macrophages, express GGT5 protein [14]. GGT5 is a cell membrane protein that hydrolyzes the gamma-glutamyl moiety of GSH and GSH S-conjugates [15]. It plays a critical role in redox regulation, drug metabolism, immune function and other processes within the body [16]. In addition, GGT5 is also able to convert leukotriene C4 (LTC4) to leukotriene D4, but appears to have distinct substrate specificity compared to GGT [17]. However, previous study suggested that GGT5 knockout mice did not show any phenotypic abnormalities unless under stress [18]. In zymosan-A-induced peritonitis model, GGT5-deficient mice cannot metabolize LTC4 in peritoneal fluid, which results in attenuated neutrophils infiltration into the peritoneum [19]. Moreover, during *Aspergillus fumigatus* induced asthma, GGT5 knockout mice have increased airway hyper-responsiveness [20]. These evidences suggest that GGT5 may promote the inflammatory response. However, the role of GGT5 in cancer progression have not yet been characterized.

In the present study, we first reported that high expression of protein GGT5 in cancer-associated fibroblasts (CAFs) predicted the poorer survival of LUAD patients. Moreover, our *in vitro* and *in vivo* functional studies indicated that CAFs-derived GGT5 promoted cell proliferation and drug resistance by reducing the reactive oxygen species (ROS) level in LUAD cells, suggesting that GGT5 may be a promising therapeutic target for patients with LUAD.

## RESULTS

### High expression of GGT5 predicts the poor outcome of patients with LUAD

**Figure 1:**
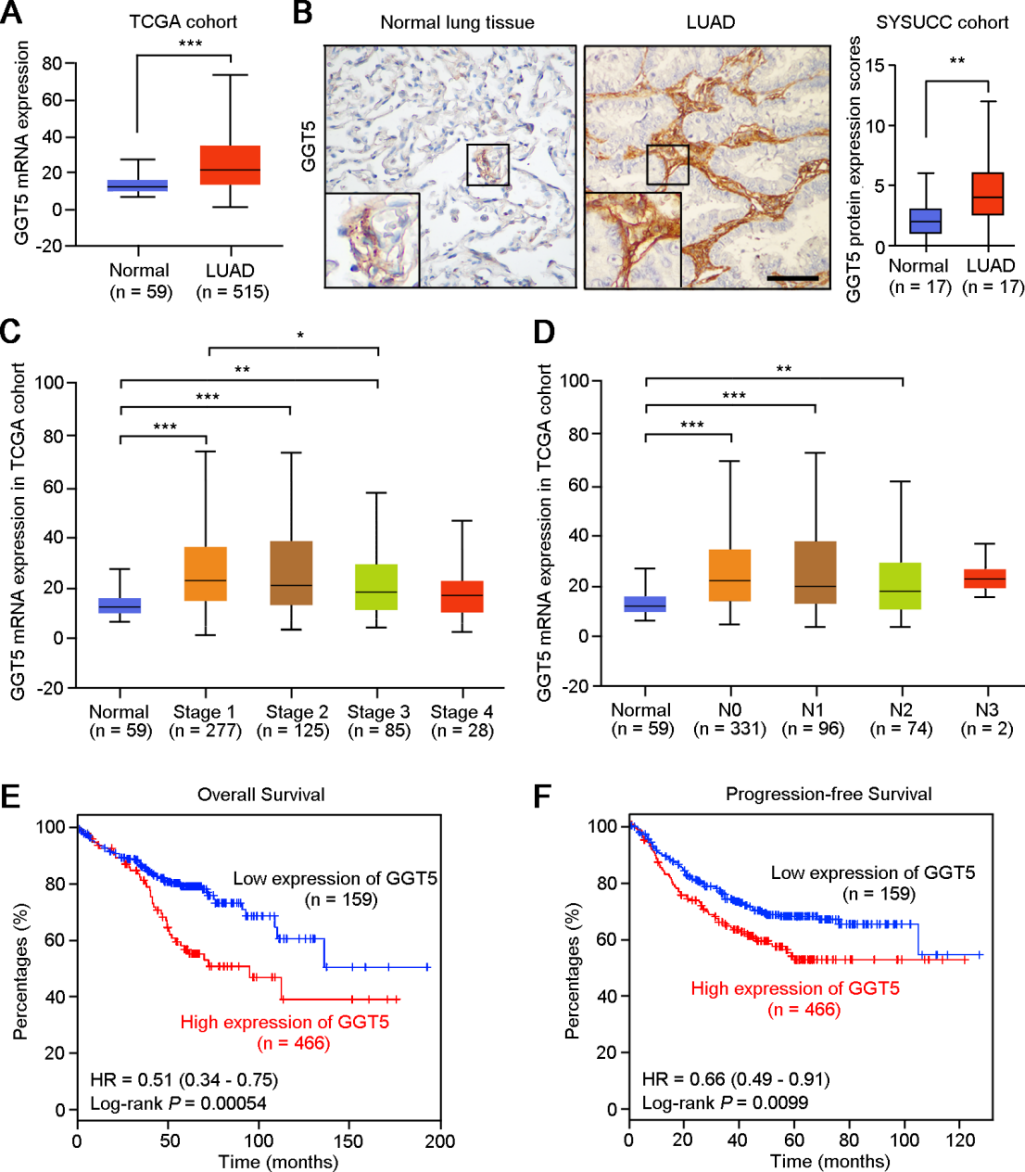
**High expression of GGT5 is associated with the poor survival of patients with LUAD.** (**A**) The mRNA expression of *GGT5* in normal lung tissues (n = 59) and LUAD (n = 515) were analyzed using The Cancer Genome Atlas database (TCGA) cohort. (**B**) Immunohistochemical (IHC) staining showed the protein expression of GGT5 in normal lung and LUAD tissues. Scale bar, 200 μm. (**C**, **D**) *GGT5* expression is related to cancer stages (**C**) and (**D**) lymphatic metastasis in LUAD. (**E**, **F**) High expression of *GGT5* predicts the poor survival of patients with LUAD. Patients with high or low expression of GGT5 were distinguished based on ROC curve analysis, and the cutoff value of GGT5 expression value was set as 19. In panels (**A**–**D**), data represent mean ± SD. *, *P* < 0.05; **, *P* < 0.01; ***, *P* < 0.001.

GGT5 is a member of the GGT family, but its role in cancer is undefined, especially in LUAD. First, Disease Ontology analysis of *GGT5* in human using Coexpedia indicated that *GGT5* was closely associated with malignant cancer development, including lung cancer (Supplementary Figure 1A). Moreover, gene expression data from The Cancer Genome Atlas (TCGA) database (Figure 1A) and Gene Expression Omnibus (GEO) cohort (GSE2514, Supplementary Figure 1B) demonstrated that *GGT5* mRNA expression was substantially higher in LUAD tissues than that in normal lung tissues. Consistent with these biostatistics, IHC staining also showed the increased protein levels of GGT5 in clinical LUAD samples than that in the paired non-tumor tissues (Figure 1B, Supplementary Figure 1C).

In addition, according to the clinical data from TCGA database, the mRNA expression of *GGT5* was up-regulated in patients with early-stage of LUAD (Figure 1C) and lymph node metastasis (Figure 1D). Survival analysis with Kaplan-Meier Plotter (http://kmplot.com/analysis/) suggested that both overall survival (*P* = 0.00054) and progression-free survival (*P* = 0.0099) of LUAD patients with high *GGT5* expression (≥ cutoff value 19) were significantly shorter than those with low levels of *GGT5* expression (< cutoff value 19) (Figure 1E, 1F). Taken together, these evidences implicate an aggressive role of GGT5 in LUAD progression.

### GGT5 is specifically expressed in CAFs, but not in tumor cells

**Figure 2:**
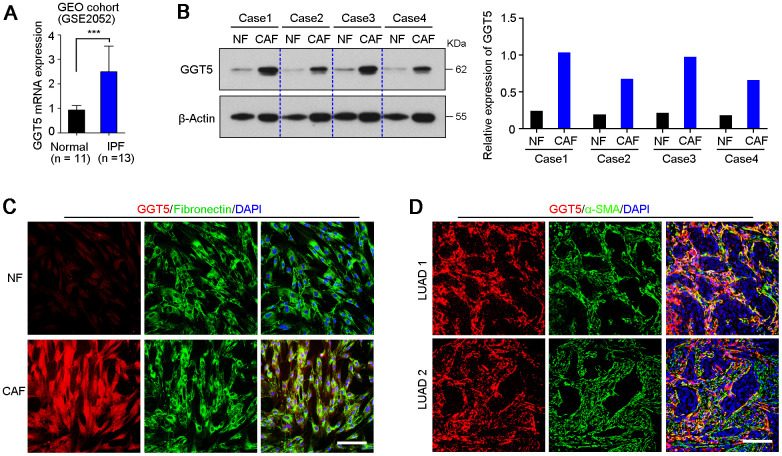
**GGT5 is highly expressed in CAFs.** (**A**) The expression level of *GGT5* in normal lung tissues and lung tissues from patients with idiopathic pulmonary fibrosis (IPF) were analyzed using GEO public cohort (https://www.ncbi.nlm.nih.gov/geo/query/acc.cgi?acc=GSE2052). Data represent mean ± SD. ***, *P* < 0.001. (**B**) Western blotting showed the high expression of GGT5 in CAFs than that in paired normal fibroblasts (NFs). β-Actin was also tested as a loading control. The relative expression of GGT5 was indicated in right panel based on the gray value of protein bands (ImageJ software). (**C**) The expression of GGT5 in NFs and CAFs *in vitro* were analyzed by immunofluorescent (IF) double-staining with antibodies against GGT5 (red) and Fibronectin (green, a marker of fibroblasts). Scale bare, 50 μm. (**D**) IF double-staining with antibodies against GGT5 (red) and α-SMA (green, a marker of CAFs) were performed on two LUAD tissue sections. Scale bar, 200 μm. In panels **C** and D, the cell nuclei were counterstained with DAPI (blue).

GEO public cohort analysis (GSE2052) indicated that gene *GGT5* was high expressed in lung tissues from patients with idiopathic pulmonary fibrosis, a lethal disorder characterized by the aberrant accumulation of fibroblasts and myofibroblasts [21], than that in normal lung tissues (Figure 2A). Moreover, our previous data of IHC staining also showed that GGT5-positive cells was specifically deposited in tumor stroma in clinical LUAD tissue sections (Figure 1B). These evidences suggest that protein GGT5 may be expressed by CAFs in LUAD microenvironment. To confirm the hypothesis, the expression levels of GGT5 in primary normal lung fibroblasts (NFs) and paired CAFs from patients with LUAD were tested by western blotting (Figure 2B) and IF staining (Figure 2C). The results displayed the increased protein expression of GGT5 in CAFs that that in corresponding NFs (Figure 2B, 2C). In addition, IF double-staining with antibodies against GGT5 and α-SMA (a marker of CAFs) in LUAD tissues showed that GGT5 was specifically expressed in CAFs, but not in tumor cells (Figure 2D).

### Interfering the expression of GGT5 in CAFs inhibits LUAD cell growth *in vitro*

**Figure 3:**
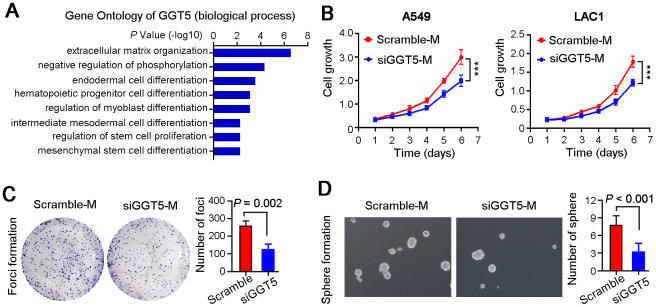
**Targeting GGT5 in CAFs decreases the LUAD cell growth *in vitro*.** (**A**) Gene Ontology Enrichment analysis (biological process) of GGT5 using Coexpedia internet tool (http://www.coexpedia.org/). (**B**) Conditioned media from CAFs transfected with scramble (Scramble-M) or siRNA targeting GGT5 (siGGT5-M) were used to treat LUAD cells A549 and LAC1, respectively, and cell growth curves were measured for six days. (**C**) Representative images of the foci formation of A549 cell in monolayer culture treated with Scramble-M or siGGT5-M. The number of foci was counted in right panel. (**D**) Representative images of the spheres formation in soft agar of A549 cell treated with Scramble-M or siGGT5-M. The number of spheres was analyzed in right panel. In panels B-D, data represent mean ± SEM. **, *P* < 0.01; ***, *P* < 0.001.

To investigate the role of GGT5 in LUAD progression, Gene Ontology Enrichment analysis (biological process) was performed using Coexpedia. Analysis results showed that GGT5 was significantly related to cell differentiation and cell cycle regulation, hinting that GGT5 might mediate the crosstalk between CAFs and tumor cells (Figure 3A). Hence, we analyzed the cell growth rate of two LUAD cell lines A549 and LAC1 treated with conditioned media from primary CAFs transfected with scramble (Scramble-M) or siRNA targeting *GGT5* (siGGT5-M). First, the qPCR was used to confirm the knockdown of *GGT5* by siRNA at mRNA level (Supplementary Figure 2A). Results indicated that down-regulation of GGT5 in CAFs attenuated the growth of tumor cell *in vitro* (Figure 3B). Moreover, foci formation in monolayer culture (Figure 3C) and spheres formation in soft agar (Figure 3D) analyses also suggested that interfering GGT5 expression in CAFs could inhibit tumor cell proliferation *in vitro*.

### High expression of GGT5 in CAFs enhances the drug resistance of LUAD cells

**Figure 4:**
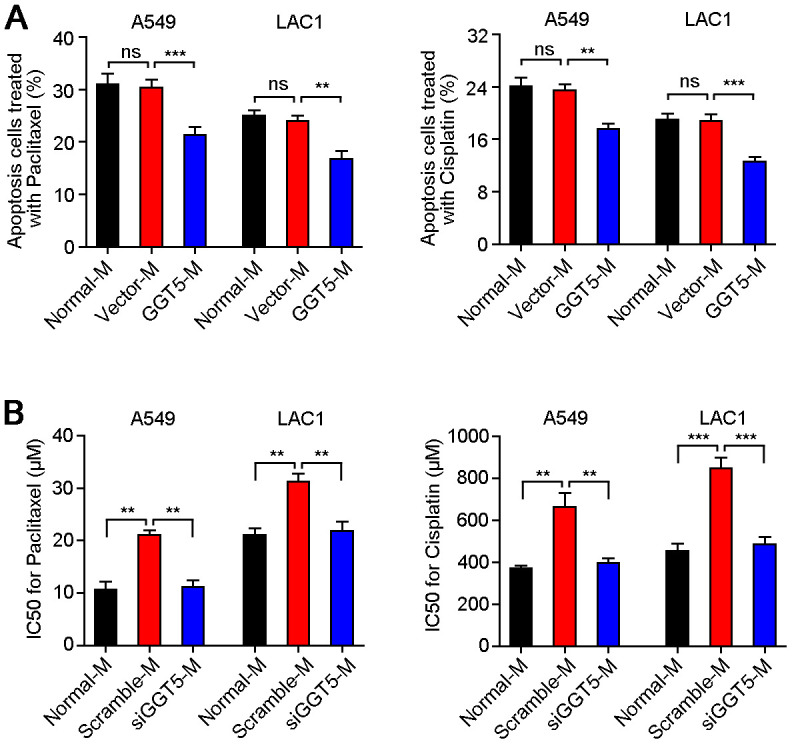
**Increased GGT5 in CAFs promotes the drug resistance of LUAD cells.** (**A**) Apoptosis cells of A549 and LAC1 cells treated with Paclitaxel (5 μM) or Cisplatin (200 μM) were analyzed under normal culture medium (Normal-M) or conditioned media from NFs transfected with vector (Vector-M) or GGT5 (GGT5-M). (**B**) The IC50 values of Paclitaxel and Cisplatin for A549 and LAC1 cells under Normal-M (DMEM + 10%FBS) or conditioned media from CAFs (Scramble-M or siGGT5-M). In panels **A** and **B**, data represent mean ± SEM. ns, no significant difference. **, *P* < 0.01; ***, *P* < 0.001.

Since it has been proved that GGT1 as a member of GGT family was involved in drug resistance [22], but role of GGT5 during cancer chemotherapy is unclear. Therefore, exogenous overexpression of GGT5 in NFs was established (Supplementary Figure 2B), and the conditioned media from NFs transfected with vector (Vector-M) or GGT5 (GGT5-M) were used to culture A549 and LAC1 cells, respectively. Meanwhile, LUAD cells were treated with chemotherapeutic drugs Paclitaxel (5 μM) or Cisplatin (200 μM). Flow cytometry tests indicated that increased expression of GGT5 in NFs reduced the apoptotic rate of LUAD cells (Figure 4A). In addition, the chemosensitivity of LUAD cells regulated by CAFs with or without interference of GGT5 was also explored *in vitro*. Results showed that down-regulation of GGT5 in CAFs impaired the chemoresistance of LUAD cells (Figure 4B).

### Increased GGT5 in CAFs reduces the ROS level in LUAD cells

**Figure 5:**
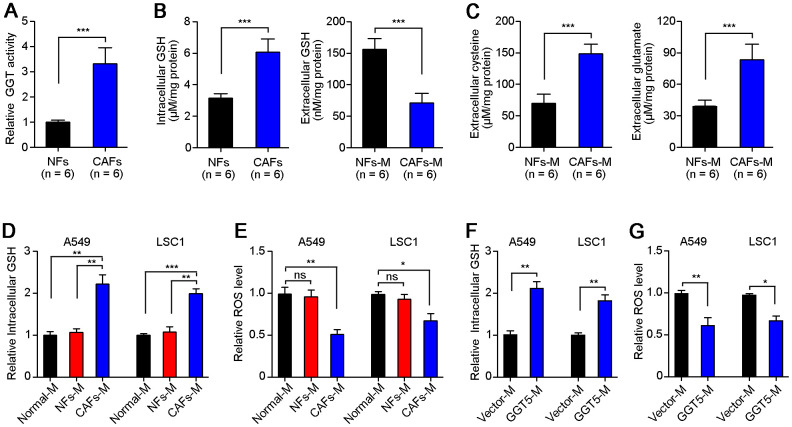
**High level of GGT5 in CAFs decreases the ROS level of LUAD cells.** (**A**) The GGT activity in paired NFs and CAFs was tested *in vitro* (n = 6). (**B**) The level of GSH in NFs and CAFs or their conditioned media (NFs-M and CAFs-M) was detected. (**C**) The extracellular cysteine or glutamate in NFs-M and CAFs-M were analyzed respectively. (**D**) The relative intracellular GSH in A549 and LAC1 cells treated with different media: Normal-M, NFs-M or CAFs-M. (**E**) The relative intracellular ROS in A549 and LAC1 cells treated with different media. (**F**) The relative intracellular GSH in A549 and LAC1 cells treated with Vector-M or GGT5-M. (**G**) The relative intracellular ROS in A549 and LAC1 cells treated with Vector-M or GGT5-M. In all panels, data represent mean ± SEM. ns, no significant difference. *, *P* < 0.05; **, *P* < 0.01; ***, *P* < 0.001.

To explore the mechanism of GGT5 in regulation of LUAD progression, we first tested the GGT activity of NFs and CAFs. Results showed that CAFs maintained the high activity of GGT than that in NFs (Figure 5A). GGT1 as a member of GGT family has been reported to promote lung cancer progression [23]. However, our IF staining showed that the expression of GGT1 in NFs and CAFs was negative (Supplementary Figure 3A). Next, we measured the GGT activity in NFs transfected with vector or GGT5 (Supplementary Figure 3B) and CAFs with or without siGGT5 (Supplementary Figure 3C). Results showed that GGT activity was increased in NFs after GGT5 overexpression and decreased in CAFs when GGT5 was silenced (Supplementary Figure 3B, 3C). Moreover, the increased GGT activity in NFs after GGT5 overexpression was inhibited by a GGT inhibitor GGsTop (Supplementary Figure 3D) [24]. These data suggested that up-regulated GGT5 was responsible for the high GGT activity of CAFs. It has been reported that GGT5 was capable of cleaving the gamma-glutamyl moiety of GSH [23]. Hence, GSH level in fibroblasts or conditioned media (CAFs-M and CAFs-M) were analyzed. CAFs synthesize more intracellular GSH compared to NFs, but less extracellular of GSH deposits in CAFs-M than that in CAFs-M, which may be due to that extracellular of GSH is cleaved into cysteine and glutamate by GGT5 (Figure 5B). Therefore, we next tested the extracellular cysteine or glutamate in NFs-M and CAFs-M, respectively. High levels of cysteine and glutamate in CAFs-M than that in NFs-M were confirmed (Figure 5C).

Next, we further analyzed the intracellular GSH (Figure 5D) and ROS (Figure 5E) in A549 and LAC1 cells treated with different cultures: Normal-M, NFs-M or CAFs-M. Unsurprisingly, CAFs-M treatment significantly increased the intracellular GSH in A549 and LAC1 cells (Figure 5D), and the intracellular ROS in LUAD cells was reduced after culture with CAFs-M, compared with NFs-M or Normal-M (Figure 5E). Most importantly, overexpression of GGT5 in NFs could also induce the high level of intracellular GSH and less intracellular ROS in A549 and LAC1 cells (Figure 5F). These *in vitro* data suggest that CAFs-derived GGT5 promotes the transfer of GSH into LUAD cells from tumor microenvironment, which further reduces the intracellular ROS level in LUAD cells.

### Targeting GGT5 enhances the chemosensitivity of LUAD cells *in vivo*

**Figure 6:**
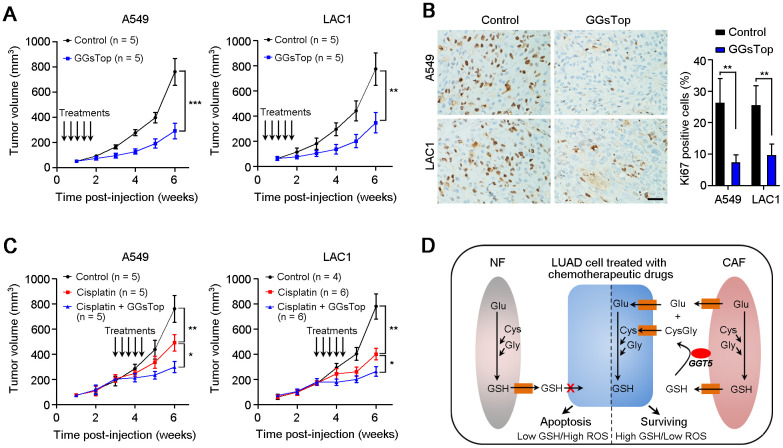
**Targeting GGT5 inhibits tumor growth and enhances the drug sensitivity of LUAD cells *in vivo*.** (**A**) Inhibiting GGT5 activity with a small-molecule inhibitor GGsTop (*i.p.* injection, 1 mg/kg) suppresses the tumor growth in nude mice xenograft model. (**B**) IHC staining with antibody against Ki67 (a marker of proliferative cell) in tissue sections from transplantation tumors treated with DMSO control or GGsTop. (**C**) Co-administration of Cisplatin (*i.p.* injection, 6 mg/kg) and GGsTop (*i.p.* injection, 1 mg/kg) significantly inhibits the tumor growth. (**D**) Schematic diagram for high level of GGT5 in CAFs contributing to LUAD cell survival and drug resistance by increasing intracellular GSH and decreasing the intracellular ROS level in tumor cells. In panels A and C, data represent mean ± SD, and in panel B, data represent mean ± SEM. In panels A-C, *, *P* < 0.05; **, *P* < 0.01; ***, *P* < 0.001.

All the above data indicate that GGT5 may be a promising therapeutic target in LUAD. Hence, we further explored the influence of GGT5 blocking with GGsTop on tumor growth and chemosensitivity in mouse xenograft model. First, inhibiting GGT5 activity with GGsTop (*i.p.* injection, 1 mg/kg) markedly suppresses the tumor growth in nude mice xenograft model in A549 and LAC1 cells and does not affect the body weight of mice (Figure 6A, Supplementary Figure 4). Moreover, IHC staining with antibody against Ki67 (a marker of proliferative cell) in tissue sections from transplantation tumors showed that treatment with GGsTop inhibited the proliferation of A549 and LAC1 cells (Figure 6B). Furthermore, co-administration of Cisplatin (*i.p.* injection, 6 mg/kg) and GGsTop (*i.p.* injection, 1 mg/kg) significantly inhibited the tumor growth (Figure 6C). Taken together, our study illuminates that high level of GGT5 in CAFs contributes to tumor cell survival and drug resistance by increasing intracellular GSH and reducing the intracellular ROS level in LUAD cells.

## DISCUSSION

In this study, we analyzed the expression of GGT5 in LUAD and normal lung tissues for the first time, and found that GGT5 was highly expressed in LUAD, which predicted the poor prognosis of patients with LUAD. Interestingly, our study clearly confirmed that GGT5 specially expressed in CAFs, but not in tumor cells, which contributed to tumor cell proliferation and drug resistance by increasing intracellular GSH and reducing the intracellular ROS level in LUAD cells. Therefore, our data revealed a novel crosstalk mechanism mediated by GGT5 between tumor cells and CAFs during cancer progression and treatments.

Recently, accumulating evidences suggested that elevated serum GGT was involved in tumorigenesis and progression, such as gastric cancer [25], colorectal cancer [26], breast cancer [10] and cervical cancer [27]. However, the expression of GGT5 in primary tumor tissues has not been reported. Hence, based on the analysis of TCGA database and GEO public cohorts, we found the high expression of GGT5 in LUAD tissues than that in normal lung tissues. Moreover, high GGT5 in LUAD tissues predicts the adverse overall survival and progression-free survival for LUAD patients. These findings suggest the aggressive role of GGT5 in LUAD progression, but the function and mechanism are not clear. One recent study showed that GGT5 highly expressed in follicular dendritic cells promoting B cell confinement to germinal centers in lymphoid tissues [28]. Our further study proved that CAFs in LUAD microenvironment were GGT5-positive, which indicated that GGT5 might mediate the crosstalk between tumor cells and CAFs. Therefore, the conditioned medium from CAFs with siGTT5 was used to treat LUAD cell *in vitro*, and results showed that knockdown of GGT5 in CAFs could inhibit the growth of LUAD cells. These data suggest that CAFs remold the tumor microenvironment promoting tumor cell growth.

It has been proved that GGT was involved in drug resistance in tumor progression [29, 30]. Herein, we also test the regulation of CAFs-derived GGT5 on the chemosensitivity of LUAD cells. Results showed that down-regulation of GGT5 in CAFs impaired the chemoresistance of LUAD cells. These data indicate that GGT5 may be a potential therapeutic target for improving the treatment of LUAD patients. Oxidative stress is implicated in cancer development and progression, and increased serum GGT is associated with the high risk of cancers [25–27]. Anti-oxidation properties of GGT may underlie etiological mechanisms leading to these adverse outcomes for LUAD patients. Therefore, we firstly tested the intracellular and extracellular GSH for NFs and CAFs. Elevated intracellular GSH in CAFs and low level of GSH in CAFs-M were observed. Knockdown of GGT5 decreases the levels of cysteine and glutamate in CAFs-derived conditioned media. These substrates are necessary for synthesis of GSH. It is known that GSH as a vital regulator of the cellular redox state is involved in the proliferation and chemoresistance of cancer cells [31, 32]. Therefore, when the conditioned media from GGT5-silenced CAFs was used to treat LUAD cells, less cysteine and glutamate were provided for GSH synthesis in cancer cells, which accounted for the slower cell growth and higher drug-sensitivity of cancer cells after treatment the cultures. In tumor microenvironment, high levels of cysteine and glutamate in tumor microenvironment indicated that extracellular GSH was cleaved by CAFs-derived GGT5. These substrates will be further transported into tumor cells for GSH synthesis. Therefore, CAFs-derived GGT5 promotes cell survival and drug resistance by reducing the intracellular ROS level in LUAD cells (Figure 6D).

CAFs as a main component of tumor microenvironment have been proved to promote tumor progression, including proliferation promotion, angiogenesis, immunosuppression and drug resistance [32–34]. Therefore, targeting CAFs may be promising therapeutic strategy for cancer patients [35]. In this study, we confirmed that blocking GGT5 with a small-molecule inhibitor GGsTop could inhibit tumor growth and increase the drug-sensitivity of LUAD cells in mouse xenograft tumor model. In conclusion, our clinical data analysis and *in vitro*/*in vivo* functional studies demonstrated the aggressive role of GGT5 in LUAD, providing a novel diagnostic or therapeutic target for LUAD patients.

## MATERIALS AND METHODS

### LUAD clinical samples and cell lines

Primary LUAD specimens and their corresponding non-tumor tissues were obtained with informed consent from patients at the Sun Yat-sen University Cancer Center (Guangzhou, China). All samples used in this study were approved by the Committees for Ethical Review of research involving human subjects at the Sun Yat-sen University Cancer Center (Guangzhou, China). LUAD cell line A549 was purchased from the American Type Culture Collection (ATCC, Manassas, Virginia, USA). The other LUAD cell line LAC1 (also known as ACC212102) were established in our laboratory [36, 37]. All cell lines were cultured in high-glucose DMEM (Gibco BRL, Grand Island, NY) supplemented with 10% fetal bovine serum (FBS, Gibco BRL, Grand Island, NY) at 37°C with 5% CO_2_.

### Immunohistochemistry (IHC) and immunofluorescence (IF) staining

In brief, tissue slides with paraffin sections were deparaffinized by xylene, rehydrated using graded ethanol and rinsed with deionized water. Endogenous peroxidase activity was blocked with 3% hydrogen peroxide for 10 min at room temperature. For antigen retrieval, slides were high-pressure-treated and boiled in citrate buffer (pH 6.0, 10mM) for 15 min. Nonspecific binding was blocked with 5% normal goat serum for 30 min at room temperature. The slides were incubated with monoclonal antibodies against GGT5 (Santa Cruz Biotechnology, #sc-20640, 1:500 dilution), Ki67 (Abcam, #ab15580, 1:400 dilution), Fibronectin (Cell Signaling Technology, #26836, 1:200 dilution) or α-SMA (Cell Signaling Technology, #56856, 1:200 dilution) at 4°C overnight in a humidified chamber. For IHC staining, the slides were incubated with horseradish peroxidase-conjugated secondary antibody and stained with the DAB substrate. The representative figures of IHC staining were captured with light microscope (Olympus, Lake Success, NY). An immune-reactivity score system was applied as described previously [38]. The percentage of GGT5-positive cells was scored as 0, <5%; 1, 5%-25%; 2, 25%-50%; 3, 50%-75%; 4, 75%-100%. The intensity of GGT5-positive staining was scored as 0, negative; 1, weak; 2, moderate; 3, strong. The total score was determined by the following formula: Staining index = positive percentage × intensity. For IF staining, the slides were incubation with Alexa Fluor 594 or 488-conjugated secondary antibodies (Invitrogen). The cells on coverslips were counterstained with DAPI (Invitrogen) and imaged using a confocal laser-scanning microscope (Olympus, Lake Success, NY).

### Isolation of fibroblasts

Primary NFs or CAFs were established in our laboratory after tumor resection. In detail, fresh LUAD tissue and paired non-tumorous lung tissue were cut into as small pieces as possible by surgical scissors in sterile culture dish, respectively. And then the tissue homogenate was transferred into sterile tube supplemented with 1ml Liberase (0.2 mg/ml, Roche, #5401119001) for enzymolysis at 37°C for 30 min. The suspension was filtered through a sterile Cell Strainer (40 μm, Corning) to collect a single-cell suspension. The filtrate was centrifuged at 1,500 rpm for 5 min at 4°C and washed twice with pure DMEM. The cell pellet was resuspended with 3 ml DMEM supplemented with 20% FBS and finally plated on 6-cm cell culture dishes. Based on the different adhesive capacity of fibroblasts and other cells, the unadherent cells (mainly tumor cells) were removed after culturing for 30 min at 37°C to obtain relative pure fibroblasts, because the adhesion time of fibroblasts is much shorter (~20-30 min) than tumor cells (usually > 1 h). Next, when the cell density reaches ~80% dish surface area, fibroblasts are further purified by short time digestion of trypsin (~30 s). The digested cells were cultured for further isolation of fibroblasts again. Finally, the pure fibroblasts are confirmed by immunostaining with antibody against Fibronectin (Cell Signaling Technology, #26836, 1:200 dilution).

### Construction of GGT5 overexpression and knockdown fibroblasts

To study the function of *GGT5* in NFs or CAFs, full-length human *GGT5* complementary DNA was amplified by reverse transcription-PCR and was cloned into pLenti6.3/V5 expression vector (Invitrogen, Carlsbad, CA). For *GGT5* interference assay, one small interfering RNA specifically targeting *GGT5* (siGGT5, 5'-CGGGGACCAGCTGGGCAGATGAG-3') and the scramble control siRNA was purchased from GeneCopoeia (Rockville, MD). *GGT5* expression vector and control plasmids, siGGT5 and scramble control siRNA were transfected into fibroblasts using the HilyMax transfection reagent (Dojindo Corp., Japan) according to the manufacturer’s instructions, respectively. The expression level of *GGT5* was confirmed by quantitative real-time PCR (qPCR) using FastStart Universal SYBR Green Master (Roche) and a 96-well Real-Time PCR Detection System (Roche). *ACTB* was used as the internal control. The primers used were as follows: *GGT5*: 5'-GTCAGCCTAGTCCTGCTGG-3' (forward) and 5'-GGATGGCTCGTCCAATATCCG-3' (reverse); *ACTB*: 5'-CATGTACGTTGCTATCCAGGC-3'′ (forward) and 5'-CTCCTTAATGTCACGCACGAT-3' (reverse). The relative expression level (defined as fold change) of the target gene (2^-ΔΔCt^) was normalized to the endogenous *ACTB* reference (ΔCt).

### Foci formation assay

Briefly, 1×10^3^ A549 cells were seeded in each well of a 6-well plate with fibroblasts-conditioned media. After a week of culture, surviving colonies (>50 cells per colony) were counted with crystal violet (Sigma) staining. Triplicate-independent experiments were performed.

### Cell proliferation assay

The Cell Counting Kit-8 (CCK-8) assay kit (Dojindo Corp., Japan) was applied to measure the cell growth rate. A suspension of 1×10^3^ A549 or LAC1 cells were planted in each well of a 96-well plate, in which 10 μl CCK-8 was added to 90 μl of culture medium. And after 2 h incubation at 37 °C, the absorbance was measured at 450 nm. Triplicate independent experiments were performed.

### Western blot analysis

Human paired NFs and CAFs were lysed with RIPA lysis buffer (Cell Signaling Technology, Beverly, MA) supplemented with cocktail protease inhibitor (Roche). After boiling for 5 min, protein lysates were separated by 10% SDS-PAGE and then transferred to PVDF membranes (Millipore, Billerica, MA, USA). The membranes were blocked with 5% non-fat milk in TBS/Tween20 (TBST, 0.05% v/v) for 1 hour at room temperature, and incubated with primary antibodies against GGT5 (Santa Cruz Biotechnology, #sc-20640, 1:2000 dilution) and β-Tubulin (Cell Signaling Technology, #2146, 1:2000 dilution) overnight at 4 °C. The membranes were washed three times with TBST buffer for 5 min per time, and then incubated with HRP-conjugated secondary antibodies for 2 hours at room temperature. The bands were visualized with the enhanced chemiluminescence (Thermo Scientific, NY, USA), and the signals were quantified by ImageJ software (http://rsb.info.nih.gov/ij).

### GSH content and GGT activity assay

The GSH-Glo™ Glutathione Assay Kit (#V6912, Promega) was used to determine the reduced GSH content in the different medium samples. γ-Glutamyltransferase Activity Fluorometric Assay Kit (#MAK090-1KT, Sigma) was used to determine the GGT activity in fibroblasts or tumor cells. Experiments were performed with biological replicates.

### *In vivo* xenograft assay

All animal experiments were approved by Animal Ethics Committee at Sun Yat-sen University Cancer Center. Five-week-old female BALB/c nude mice were purchased from the Guangdong Medical Laboratory Animal Center (Guangzhou China). Different numbers of LUAD cells (A549: 2×10^6^ cells; LAC1: 4×10^6^ cells) in 100μl phosphate buffered saline (PBS) were injected subcutaneously into nude mice. After tumor cells was injected, GGsTop (1 mg/kg, APExBIO, #B5602) or Cisplatin (6 mg/kg, Selleck, #S1166) was administered intraperitoneally every two days for total five times. The length (L) and width (W) of tumor were measured every week by calipers, and tumor volumes were calculated as volume (mm^3^) = L × W^2^ × 0.5.

### Statistical analysis

SPSS version 17.0 (Chicago, IL) was used for all data analyses. An independent Student *t* test was used for continuous data. The mRNA expression of *GGT5* in LUAD and normal lung tissues were obtained from TCGA database via a web server UALCAN (http://ualcan.path.uab.edu/analysis.html). Survival curves were obtained from Kaplan-Meier Plotter (http://kmplot.com/analysis/). Disease Ontology and Gene Ontology analyses were based on Coexpedia (http://www.coexpedia.org/). Results were considered statistically significant when *P* < 0.05.

## Supplementary Material

Supplementary Figures
