# Supplementary material for: Cancer-associated fibroblasts-derived gamma-glutamyltransferase 5 promotes tumor growth and drug resistance in lung adenocarcinoma
Source: Aging (Albany NY). 2020 Jul 8;12(13):13220–33. doi: 10.18632/aging.103429 (PMC7377883; doi:10.18632/aging.103429)
Supplement: Supplementary Figures [file aging-12-103429-s001..pdf]

SUPPLEMENTARY FIGURES

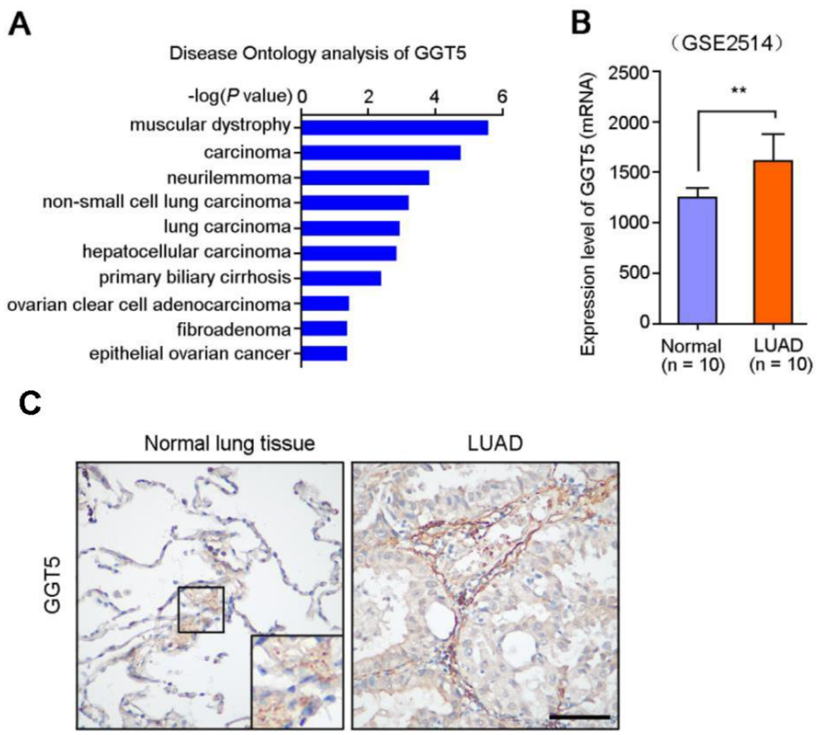

Supplementary Figure 1. High expression of GGT5 is associated with carcinogenesis. (**A**) Disease Ontology analysis of GGT5 in human using Coexpedia (http://www.coexpedia.org/). (**B**) Gene expression data from GEO cohort (GSE2514) showed the high expression of GGT5 expression in LUAD tissues than that in normal lung tissues. **, $P < 0.01$. (**C**) Immumohistochemical staining showed the protein expression of GGT5 in normal lung and LUAD tissues. Scale bar, 200 µm.

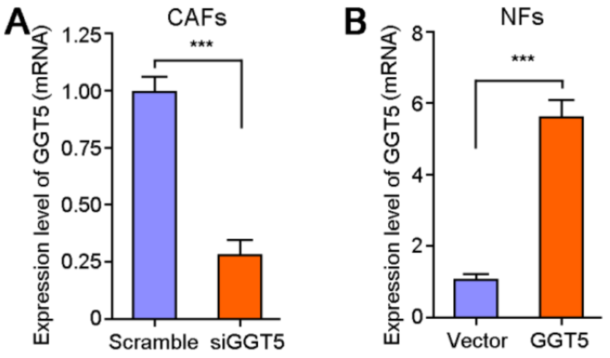

Supplementary Figure 2. The expression levels of GGT5 in CAFs after GGT5-knockdown (**A**) or in NFs with exogenous overexpression of GGT5 (**B**) were tested by qPCR. ***, $P < 0.001$.

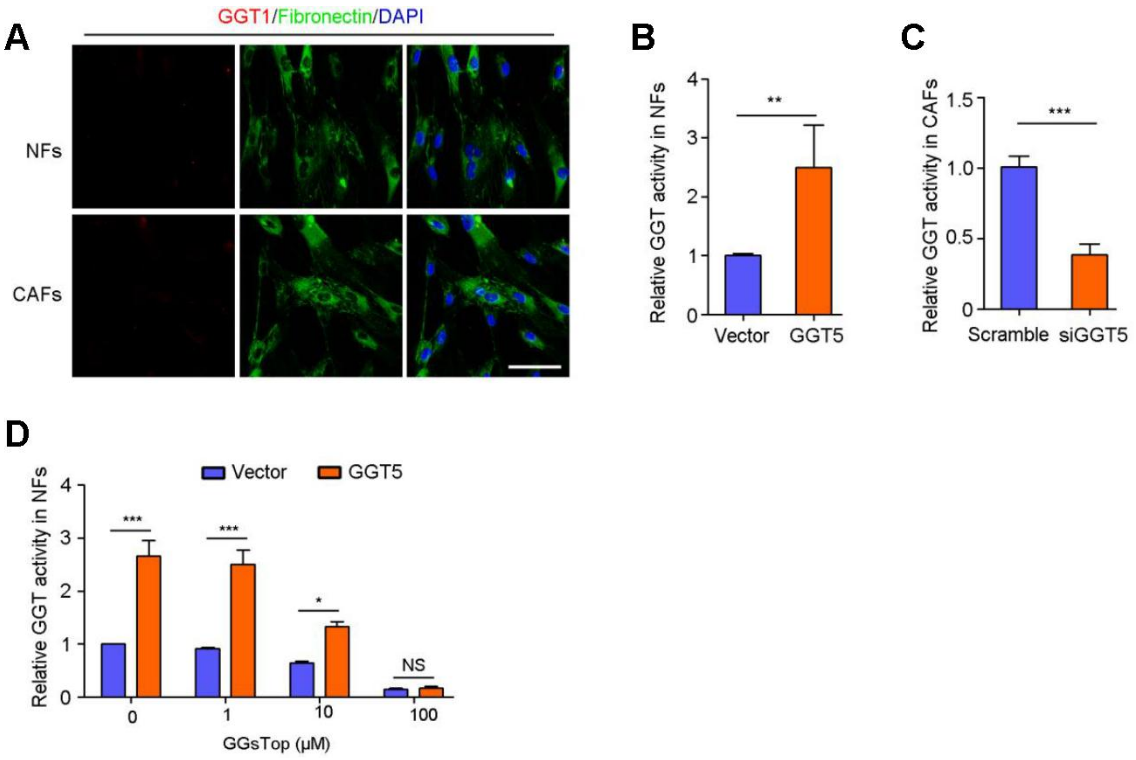

Supplementary Figure 3. Overexpression of GGT5 is responsible for the high GGT activity of CAFs. (**A**) Immunofluorescent staining in NFs and CAFs with antibodies against GGT1 (red) and fibronectin (green). Cell nuclei were counterstained with DAPI (blue). Scale bare, 50 μm. (**B**, **C**) GGT activity was increased in NFs after transfection of GGT5 (**B**), and inversely, GGT activity was decreased in CAFs when the GGT5 was silenced (**C**). (**D**) Different concentrations of GGsTop was used to treat NFs that have been transfected vector or GGT5. GGT activity was measured after 24 hours of treatments. In panels B-D, *, $P < 0.05$; **, $P < 0.01$; ***, $P < 0.001$. NS, no significant difference.

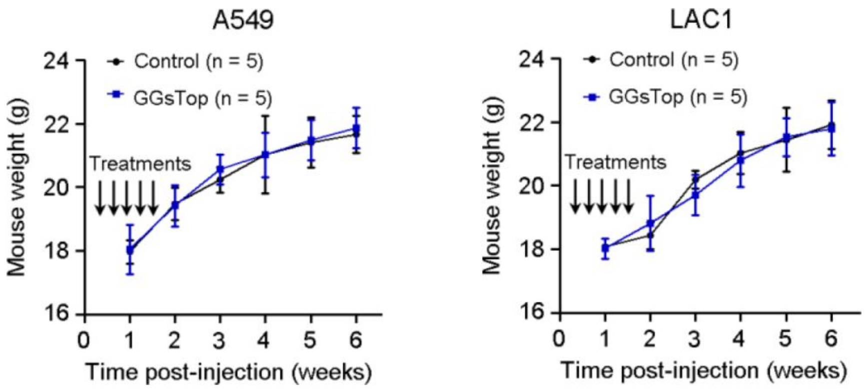

Supplementary Figure 4. Mice weights during and post- treatments.
